# Patient Dossier: Healthcare queries over distributed resources

**DOI:** 10.1371/journal.pcbi.1007291

**Published:** 2019-10-17

**Authors:** Miguel Vazquez, Alfonso Valencia

**Affiliations:** 1 Barcelona Supercomputing Center (BSC), Barcelona, Spain; 2 Department of Clinical and Molecular Medicine, Faculty of Medicine and Health Sciences, Norwegian University of Science and Technology, Trondheim, Norway; 3 ICREA, Pg. Lluís Companys, Barcelona, Spain; Queen's University, CANADA

## Abstract

As with many other aspects of the modern world, in healthcare, the explosion of data and resources opens new opportunities for the development of added-value services. Still, a number of specific conditions on this domain greatly hinders these developments, including ethical and legal issues, fragmentation of the relevant data in different locations, and a level of (meta)data complexity that requires great expertise across technical, clinical, and biological domains. We propose the Patient Dossier paradigm as a way to organize new innovative healthcare services that sorts the current limitations. The Patient Dossier conceptual framework identifies the different issues and suggests how they can be tackled in a safe, efficient, and responsible way while opening options for independent development for different players in the healthcare sector. An initial implementation of the Patient Dossier concepts in the Rbbt framework is available as open-source at https://github.com/mikisvaz and https://github.com/Rbbt-Workflows.

## Introduction

### Data in medicine

Data that can be relevant to healthcare are ubiquitous, extensive, and extremely valuable. This is due to several factors: the dramatic reduction in costs of next-generation sequencing (NGS) and its increasing scope of application, which has helped “omics” penetrate strongly into clinical practice [1–3]; the adoption of electronic health records (EHRs), which are becoming a vehicle to share the clinical histories of patients in a homogeneous and interoperable way [4,5]; and the increasing use and connectivity of devices such as medical monitors and personal wearables, which are recording biometric and lifestyle information about patients as well as the general population [6,7]. Like many other areas of the modern world, the advances in data-centric services—articulated around big data, deep learning, artificial intelligence, increased computation power, and more sophisticated approaches to managing data and computational infrastructures—are exerting a phenomenal transformative force on healthcare.

### Permed in cancer research

One of the flagships of data-centric healthcare is personalized medicine (PerMed), which holds the promise of realizing better health outcomes for patients by customizing treatment to their individual characteristics [8–10]. Genomics have brought us numerous examples of PerMed, especially in its application to cancer. For instance, the initial understanding the molecular mechanisms of tumorigenesis down to the effect of individual mutations enabled the development of targeted therapies that have made significant progress eliciting tumor remission. Even if new challenges of acquired resistance or clonal heterogeneity have emerged, the current developments clearly show the importance of precise molecular information. Indeed, in the field of cancer, a number of additional genomic applications are being developed to consolidate the initial breakthroughs of targeted therapies, opening treatment avenues like immunotherapies or combinatorial therapies [11,12]. Furthermore, the variability of drug response in terms of efficacy or toxicity can often be explained by genomic differences. For instance, the drug warfarin, a medicine that acts as an anticoagulant, shows extreme differences in the magnitude of its effect, with severe health consequences, owing to particular genomic variants in the patient [13].

### Other uses of healthcare data

Data analytics is influencing other areas of healthcare beyond PerMed. With the recent advances in artificial intelligence and personal assistants, holistic lifestyle data could be used to provide customized counselling not only to patients but to the general public regarding day-to-day lifestyle choices [14]. Wearable medical monitors could anticipate clinical episodes and alert the patient or first responders, and the process of recruitment for medical studies and clinical trials could be facilitated by functionalities such as obtaining consent and processing information with the highest guarantees; at a different scale, population-level biometrics or lifestyle changes could inform policy decisions by healthcare agencies [15]. In other words, the accessibility of patient information is making possible the creation of new high-value services. However, tapping the full potential of data analytics in healthcare remains elusive. The wealth of heterogeneous information scattered across distributed data sources (genomics, clinical histories, lifestyle, environment, etc.) needs to be gathered, integrated, and built into value-added services. Developing effective systems for handling all of that highly valuable information faces important challenges related to the privacy of the data.

### Privacy issues

Data are produced, managed, and owned by different entities: hospitals, clinics, companies, or the users themselves. Exploiting these data needs to meet strict guarantees in terms of privacy and security for the patient, as well as intellectual property and consent of use. The General Data Protection Regulation (GDPR), put forward by the European Union and assimilated by other entities around the world, faces these challenges by providing guidelines that all data-centric services must follow, including those in healthcare.

### Technical challenge

Beyond the data security issues, the nature of the different data types poses very specific technical challenges. For instance, omics data often require dealing with NGS reads, which can be extremely bulky and challenging to move around; they are also very sensitive to reidentification attacks that could pose severe threats to patient privacy [16,17]. Wearable devices such as medical monitors provide streams of data that may need be analyzed on the fly to provide real-time responses [18,19]. Managing streaming data this way challenges the habitual infrastructure setups. Data in EHRs and other medical documents are often not fully homogenized into standard terminologies, and actually, often, the most interesting information is expressed in a free-text format that needs to be processed using natural language processing (NLP) techniques [20,21].

The combination of security and privacy regulations, infrastructure challenges, and the challenges in data analysis methods themselves are crucial obstacles, especially in building healthcare services to exploit patient data in a holistic way.

Here, we propose a new conceptual framework to structure the different operations involved in bringing forward these services safely, reliably, and efficiently. We describe the Patient Dossier as a new organizing concept to tackle all these challenges.

## Outlook of future applications

The concept of Patient Dossier aims at fulfilling the applications, features, and functionalities that we foresee as critical for the healthcare future.

- Data from the patient’s clinical history will be more accessible, allowing a better engagement of the patients with healthcare services across regions and administrations.
- Proposals of personalized treatment will be facilitated thanks to the availability of quality bioinformatics recipes.
- The hassle involved in moving around large datasets and finding the right expertise to process them, such as NGS from healthcare providers, will be improved to help these technologies penetrate further in the healthcare system.
- The analysis of healthcare data on behalf of the patient will be improved by opening these analyses to the other parties, including industry and public health systems, thanks to the safety guarantees provided by the technological aspects of the framework and its compliance with legal frameworks such as the GDPR.
- Patients will become managers and controllers of their own data, based on the confidence in their engagement with the healthcare data economy with a good level of trust and accountability, thanks to the standardization and simplification of the data handling mechanisms.
- Population-level analysis can be built through the Patient Dossier if the necessary mechanisms to manage consent are set in place. The results of a population-level analysis might be aggregate statistics across the population, which the interested parties will receive without the need to have access to the individualized information.
- Indeed, a number of advances are already in progress that should help find solutions to the main technical challenges:
  ○ The regulatory framework is now being defined thanks to the GDPR; the technical support for these operations is in place thanks to the expansion of cloud providers, workflow enactment systems, and containerization systems.
  ○ International efforts, such as the Global Alliance for Genomics and Health (GA4GH), are addressing normalization aspects essential for the integrated functioning of the system, while in the numerous projects on cancer, aging, rare diseases, or drug synergies, academia and companies are joining forces to develop analysis methodology and strategies.
  ○ Large-scale infrastructures, i.e., European Bioinformatics Infrastructure (ELIXIR), or community efforts such as Galaxy (https://galaxyproject.org), are dedicated to maintaining repositories of safe and vetted software recipes and workflows, making them easily accessible, as well as to developing systems to make data findable, accessible, interoperable and reusable (FAIR) [22].

However, the availability of data is still problematic, because it is not obvious how these data are localized, especially when the question involves data across different providers. Questions such as assessing whether data are relevant, whether data are up-to-date, or whether there are associated legal and ethical issues add additional challenges. Additionally, when medical questions involve processing data across multiple sites, a mechanism to orchestrate this deployment is required, whether it is entirely distributed through a peer-to-peer communication over federated entities or requires some level of centralization.

## Patient Dossiers

Currently, services around medical data are focused on particular datasets, for instance, variant analysis for NGS data; sharing of EHR and other medical documents between hospital departments; and weight, activity, and biometric data analysis for sports monitoring wearables. The mechanisms to build high-value services on top of these, potentially incorporating information from each, are hindered by technical and ethical issues but most of all, in our opinion, by the lack of a simple yet ambitious vision of how these services could be described and realized.

### Direct questions as the core of medical services

Specific medical questions are the logical starting point to nucleate and build patient-specific services around. For example, has the patient received a particular treatment? Is he/she actively exercising? What was his/her blood pressure on the last check-up? Does the patient harbor a particular germline variant? When designing services, it would be convenient to think in terms of these questions and not to be concerned with the details about where this information resides or how it is computed. At the same time, it would be good to have some certainty that when the answer to these questions involves computational pipelines, such as when characterizing a patient genotype, the required pipelines follow guidelines that ensure good quality and homogeneous, comparable, and interoperable results.

### Defining the Patient Dossier

We define the “Patient Dossier” as the collection of particular questions that can be asked (and answered) about a patient at the current time. These questions can be very diverse, but they all should be able to be resolved by following some recipe, which will take into account the particularities of each case, such as what data are available. Usually, bioinformatics tools and pipelines focus on processing, filtering, or inferring information out of data; they can often be thought of as ingesting files in one format and producing a file in a different, more distilled format. For instance, an NGS pipeline would take files in FASTQ format and render a variant call format (VCF) file. The Patient Dossier bridges the gap from these pipelines to an application setting, for instance, when a clinician asks whether the subject has a particular variant that can help explain his or her condition.

The Patient Dossier focuses on questions that a “human” operator might ask (e.g., a clinician, a government agency, the patients themselves) as opposed to the more technical questions software tools would use to communicate, such as the availability of a given type of data for a given patient or the format in which it is encoded. These later questions belong to how the Patient Dossier is implemented but not to its interface. The questions defined in the Patient Dossier provide a language that can serve as a point of understanding between pipeline developers and the final users, sparing the latter the need to get involved with the more technical aspects. Complex queries involving different aspects of the patients’ health can be answered by composing more basic queries from the Patient Dossier.

### Simple Patient Dossier queries

Some questions just involve simple queries, but how these queries are made may differ from case to case. For instance, the answer to the question of the blood pressure on the last check-up when issued within a hospital unit might involve a query to the hospital information system, whereas when issued by a primary care system, it might involve accessing the information system on the clinic where the patient took the test or even some intermediate data repository where the user manages his or her medical information. Data need to be accessed through interfaces or “drivers” that are able to communicate with the different data providers. This is perhaps the most critical aspect of realizing the Patient Dossier and where the GDPR is more relevant.

### Security and ethical issues in the Patient Dossier

From an ethical and legal standpoint, issues of data security, patient confidentiality, and consent of use also differ from question to question and from environment to environment. The Patient Dossier provides the right level at which to describe and resolve these ethical and legal issues. Each question in the dossier should explicitly state the information it delivers, which is the subject of these considerations, regardless of the data that are used to source it. Consider, for instance, the question of whether a particular lung tumor is driven by *KRAS* mutations, a question which might be answered from some exome sequencing that was performed on a tumor biopsy. The entire data set of NGS reads might be subject to strict access rules, because even when anonymized, it could still be abused to reidentify the subject, whereas the interpretation about what drives the tumor, based on a bioinformatics recipe, would not be subject to such strict regulations because it does not pose such a direct risk to patient’s rights.

### Provenance and data economy

The Patient Dossier should not only address the issues of data security, patient privacy, and consent, it must also maintain the links on data provenance and ownership across the different transactions and transformations, i.e., what data are owned by whom and how information derived from it can be used for different purposes by third parties. When decisions are made based on analysis of data, detailed provenance describing how the analysis is conducted provides assurances, accountability, and insight. Services can be built around the availability of the data but also around how they are processed. In this sense, the Patient Dossier will be a central piece of the data economy based on patient health data.

### Computing, pipelines, and high-performance computing

Some of the questions in the Patient Dossier will involve expensive calculations. Coming back to our example of the *KRAS*-driven lung tumor, the computations involved in figuring out what drives a tumor from the exome sequencing NGS reads poses a significant computational challenge. Not only are significant computational resources required but also an important level of technical expertise. How these data are analyzed resembles laboratory protocols: it’s a recipe composed of a series of steps that are designed to ensure reliable results.

By focusing on the medical questions and how they are answered as opposed to focusing on the data files themselves (see Table 1), the Patient Dossier will become the place to organize how these recipes are maintained and made accessible to the different parties. Maintaining these recipes so that they meet with best practices in each field and incorporate the latest technological advances is a task that should be assumed by experts. Likewise, maintaining the computational infrastructure to enact these recipes should be entrusted to specialized entities, such as cloud providers and HPC centers. In other words, to handle the systems for accessing data, the Patient Dossier must rely on trustworthy and secure infrastructure to perform these computations with the highest guarantees, involving the adequate software recipes and computational resources able to prevent leakage of sensitive data and hardened against attacks by malicious parties.

**Table 1:** Broad differences between the Patient Dossier and the current paradigm.

	Current paradigm	Proposal for the Patient Dossier
**Basic object**	Data	Medical questions
**Processing workflows**	From file format to file format	Medical question to question
**Data processor**	On site of the user after gathering data, or using a limited predefined set of processes on the data provider	Distributed or brokered, guided by the granularity of the questions and how they compose
**Exploitation**	Authorized data access	Authorized question access

## Implementing the Patient Dossier

The distributed scenario described previously requires implementations of the Patient Dossier to be modular and adapted to the peculiarities of each different software environment. We believe that an approach not tied to a single technical implementation of the Patient Dossier will help adoption and should spark the development of a variety of solutions, sorting out the specific implementation practicalities in each environment. We will discuss our own implementation of the Patient Dossier in a following section, but we would like to emphasize that our definition should be general enough to encompass, to some extent, the mechanisms that are already in place at many PerMed initiatives. Having said that, there are three things that we believe are important to implementing a Patient Dossier and from which we would like to extract some general recommendations.

### Recipes as workflows

Workflows are increasingly popular in bioinformatics, with many alternative software tools to implement and enact them. Some popular projects in workflow community are Nextflow (https://www.nextflow.io/), Galaxy, common workflow language (CWL), and workflow definition language (WDL). Workflows commonly incorporate the idea that results get built in successive steps, each step being a refinement of a previous one. This idea of progressively building the final result through a sequence of steps is not new to informatics, because, for example, it is the basis of the software compilation tool “make.” Just like a compiled application binary developed in C gets progressively built from its smaller constituents, a general question to the Patient Dossier will be built through a series of steps and implemented as a recipe in a workflow enactment tool. The parallel between the “make” tool and general workflows has been noted elsewhere, for instance, in the Snakemake tool (https://snakemake.readthedocs.io).

**Fig 1:**
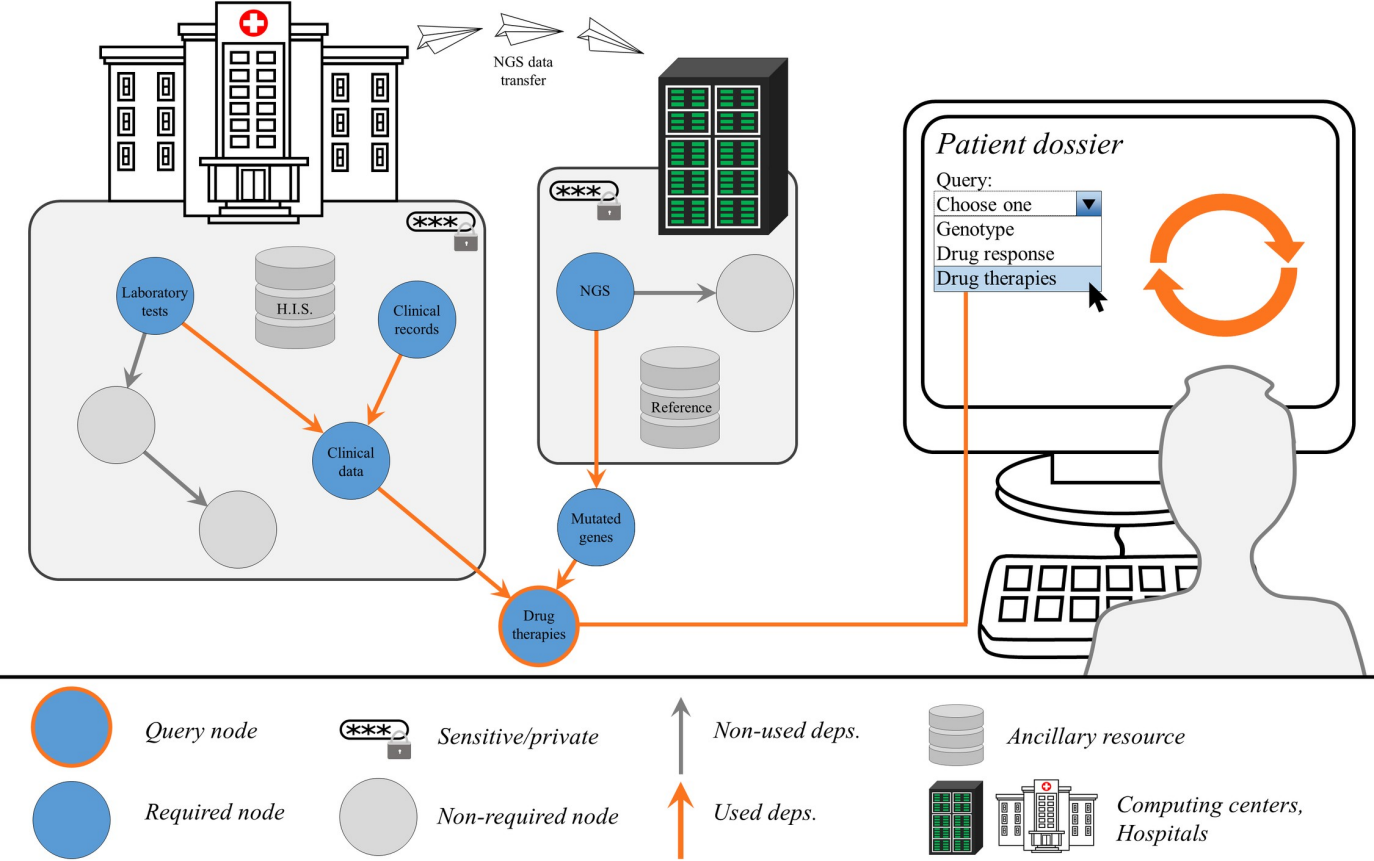
Example query over the Patient Dossier. The user, a clinician in this case, interrogates the Patient Dossier of a cancer patient for the recommended therapies, which is a particular node in the dependency tree. This, in turn, enacts a cascade of computations, because computing a node requires that other nodes be available. The arrows between nodes represent information flowing and being transformed or processed by the different recipes; for instance, NGS reads get transformed into mutated genes through variant calling and annotation pipelines. In this particular example, the recommended therapies result from considering clinical data and the list of mutated genes. Clinical data get composed inside the hospital through queries to the HIS, including clinical history and laboratory tests results. The image shows a single hospital, but data could potentially be aggregated from several hospitals and healthcare centers. The NGS data in this example were transferred to a computing center that took care of calculating the mutated genes. Sensitive information resides in silos and may only be gathered under strict access controls, such as clinical information or NGS reads. Nonsensitive information, such as the list of mutated genes or the set of drug therapy recommendations, might be accessed more widely through more lax access controls (provided the patient information is anonymized), for instance, in population-level queries issued by a different user, such as a government institution deciding on medical spending policy or a pharma company designing a clinical trial. HIS, hospital information system; NGS, next-generation sequencing.

The Patient Dossier can then be grown out of data through the use of software recipes or other smaller workflows. A convenient way to visualize a workflow that answers to Patient Dossier questions will be a dependency tree like the one in Fig 1. Each node of the dependency tree is a step in the workflow and represents a particular piece of data or information. These nodes can be seen as Patient Dossier queries themselves with associated considerations of privacy and computational costs. Different nodes in the dependency tree can be reused for different purposes, as shown in Fig 1. There is no need to enact all possible computations to generate all the nodes in the graph, just what is required. Nodes might be already available if they were required before or if these were precomputed as part of a data management policy. All nodes in the graph in principle could be Patient Dossier queries, yet most likely some would represent intermediate processing steps with no interest for the final user. Being able to trace back a particular result through the dependency tree allows checking its provenance when making important decisions.

### Access to data and computing infrastructure

Not all the steps involved in resolving a Patient Dossier question need to be enacted in the same location. Nowadays, the penetrance of virtualization and containerization technologies allows the deployment of computations across the entire “computing continuum,” from edge devices to cloud infrastructures and HPC resources. This distributed setup helps circumvent many of the issues regarding data security and computational costs. For instance, a data controller that holds very sensitive data may be able to export a safer version of these data in the form of a distilled result through Patient Dossier recipes; this way, sensitive data never leave the site. Likewise, sensitive data might travel through secure channels to a computational resource, where they might collate with other sensitive data, to be processed by analytical methods, and the final result delivered to the interested party; this way, there is no need to trust the interested party with the sensitive data in the first place. The computational resource should thus offer a hardened environment, perhaps disconnected from the internet and with ephemeral storage, to ensure the highest guarantees protecting the patient data. Thanks to this enhanced trust, medical devices and wearables used by patients will be able to provide timely advice and health alerts or require medical assistance based on artificial intelligence models running over constant streams of biometrics data without security or privacy risk.

### Scheduling and data management policies

The way to answer a medical question through the Patient Dossier is to follow a defined step-by-step recipe, which lays out how to process the data to arrive at that answer. However, the entire recipe does not always have to be run at the time each question is formulated; complex steps may be planned in a more appropriate way to prepare for future questions. For example, when NGS data arrive at a compute facility, the policy of the center might dictate that the alignment and variant calling tasks be scheduled immediately and plan how and for how long the resulting files should be stored. These are realistic requirements, and the Patient Dossier does not challenge them; it only orders them and makes them as explicit as possible in the form of code (e.g., workflows). Some steps might involve a certain level of manual curation or quality control; these need to be incorporated in the Patient Dossier as part of its code of instructions in a way that will make it possible to preserve the provenance of the results and reproducibility of the process. In summary, the Patient Dossier is designed to make the process of answering complicated medical questions as seamless as possible, but it is also called to help manage the complexities of modern biomedical data analysis by accounting for them explicitly in its recipes.

### The development ecosystem

Progress in delivering a new type of value-added healthcare services would be a collective effort, so it is important that the Patient Dossier opens up participation to different players. Our view of the Patient Dossier is that it must have roles for providers of computational resources, data, secure data transmission mechanisms, analytical methods, and ancillary data resources that help cast the patient data into context. The distributed nature of the Patient Dossier should allow the protection of intellectual property while still maintaining provenance and accountability information. A responsible and accountable engagement from all parties, including industry, will promote innovation across a wide array of services catering to the healthcare providers, administration, and patients. The development of software tools around analytical methods, workflow enactment, and infrastructure management is currently very active and progressing in huge strides, not only in the medical and bioinformatics communities. This reaffirms our idea that the implementation of the Patient Dossier should not be tied to a specific implementation, at least for the time being, but rather a wealth of different solutions should be fostered to match the previously mentioned requirements on flexible scheduling and integral data management.

### Proof of concept: The implementation of the Patient Dossier in the Rbbt framework

We have implemented the initial ideas behind the Patient Dossier in the workflow enactment tool Rbbt (Ruby Bioinformatics Toolkit). The implementation includes the following:

- Building of results through a cascade of steps that form a dependency tree and the modular architecture of functionalities, which can be manipulated independently. In Rbbt, task-specific workflows can be composed into larger workflows answering arbitrarily complex questions formulated in the Patient Dossier.
- Rbbt workflows, or parts of them, can be served through remote servers via REpresentational State Transfer (REST) interfaces facilitating the deployment of complex workflows leveraging functionalities that require specialized resources, require complicated setups, or are managed by parties that wish to protect their intellectual property.
- Cloud or HPC resources can be directly used to deal with larger workloads. These alternative deployment options can be configured transparently while maintaining provenance information. For example, in the implementation of Rbbt for Pan-Cancer Analysis of Whole Genomes (PCAWG), PCAWG-Scout [23] (the server exposing the functionalities to the public) does not hold any sensitive data; instead, whenever it needs to build a result that requires these sensitive data, it relays those steps to a second server, locked behind an institutional firewall, which holds secure data and only serves back “safe” analysis results.
- Workflows are broken down into thematic workflow modules that can be combined to allow a flexible deployment of computations and data. In Rbbt, this approach is used to deal with questions involving privacy issues or large data size and costly processing workloads, like when dealing with NGS data. The modular approach also allows workflow modules to be maintained by different teams.
- The Rbbt workflow enactment tool allows the use of configuration options that are used by different steps across the dependency tree, and it implements a strategy for the reuse of these intermediate results, accounting for the configuration options and the step they are used in. This allows an efficient exploration of configuration options, which can be helpful to validate and double check results. For instance, the Rbbt genomic pipeline can be run entirely over different genome builds, using different variant callers or different sets of filters to evaluate how these affect the results.
- The implementation of the Patient Dossier in Rbbt is prepared to automatically save detailed provenance of the complete process, including the configuration options used at each step, avoiding registering omissions and errors when imputing these metadata.
- Updates of the results are triggered by the Rbbt framework if the underlying data dependencies are updated, ensuring consistency. The complete dependency graphs can be grafted from one system to another without breaking the provenance structure, building the foundation to implement efficient data management and archiving strategies. For example, sensitive parts of workflows—for instance, performing somatic variant calling—can be enacted in hardened environments, and the portions of the dependency graph that are saved can be grafted onto a different location to enable a whole other set of downstream analyses, such as determining tumor clonal evolution or its mutational signatures.
- The Patient Dossier requires a querying interface, and Rbbt implements these functionalities through an expressive interface supporting command-line, HTML, or programmatic operations.

To illustrate how the Patient Dossier concept has been mapped into the Rbbt, let us consider the case of genomic analyses. The Rbbt offers the “Sample” workflow module, which is a repository of many questions around molecular data from patients. Questions typical of the Patient Dossier include what tumor suppressors and oncogenes have their function potentially disrupted by mutations, which genes can be considered (possibly) totally nonfunctional (e.g., a damaging mutation combined with a loss of heterozygosity due to copy number variation (CNV) or a combination of two damaging mutations that could affect both alleles), which transcription factors and signaling molecules seem to be active in the tumor, or what drug treatments can be recommended or not to the patient based on this information. The Rbbt Sample module leverages other workflows that perform more specific tasks. For instance, the “Sequence” workflow module translates genomic mutations into protein mutations and finds mutations over splicing sites or over regulatory regions.

Examples of Rbbt-implemented workflow modules that can address standard Patient Dossier questions include the following: the Sequence module, in which specific tools can be selected (such as the popular ANNOVAR [24] or variant effect predictor [VEP] [25]); the “Structure” module, which calculates distances in the Protein Data Bank (PDB) structures of proteins to find mutation clusters or mutations falling over regions of interest or in their close proximity; and the “Mutation Signatures” module, which determines the mutation signatures likely to have induced these mutations.

The Sample module has mechanisms to find the necessary information in specific locations in the file system, but these mechanisms can be overridden to accommodate the details of different datasets; for instance, for queries on large datasets (such as the International Cancer Genome Consortium [ICGC] data), the mutations can be gathered first from the ICGC data hub and stored in the file system in the correct format. User’s datasets, in the form of FASTQ files, are processed into VCF files using the high-throughput sequencing (HTS) workflow module, following best practices defined in the community and using the appropriate auxiliary data, such as the correct genome build, panel of normals controls, or the appropriate interval files for the capture technology use. For the processing of NGS reads, the mechanisms issue the work to the appropriate HPC environment, where the bulky and highly sensitive data are subject to appropriate data management policies defined by the specific institutional data management policies.

The Rbbt framework and all the workflow modules discussed here are open-source and can be found in GitHub (https://github.com/mikisvaz and https://github.com/Rbbt-Workflows). Rbbt has been developed with internal use in mind, so some expertise is required to set it up and adapt it to the details of a particular infrastructure or a particular project. The more proficient reader might be able to try it out, at least in part, with the help of the Rbbt documentation (http://mikisvaz.github.io/rbbt/); a good place to start is the ICGC workflow module.

The Rbbt framework is a versatile and powerful tool for developers of methods, pipelines, and infrastructure who know how to use it. It exemplifies some of the core elements in the Patient Dossier, but—in addition to the fact that the documentation is too lacking for general adoption, at least at the time of this writing—it still falls short of implementing the complete picture. Its functionalities cover mostly NGS analyses, which are a particular part of the Patient Dossier but not the only or necessarily the most interesting one. The ability to include information from wearables and lifestyle is among the most promising features of the new paradigm, and for these specific features, work needs to be done, in particular, to gather data from such resources. Rbbt is prepared to work with cohorts but has no mechanisms to deal with ad hoc, on-demand, population-level analyses; for that, there needs to be mechanisms to identify these potential subjects, obtain consent, etc. Some of these questions will be addressed by metadata policies, such as the ones that inspired the FAIR principles; others will be worked out as the GDPR develops. Finally, the Rbbt does not currently exemplify enough the harmonized use of results stemming from different patient data sources, for instance, NGS and wearables. In this regard, Rbbt has been used to simulate the signaling networks of cell lines, for which cell-line data were used from different resources such as Cancer Cell Line Encyclopedia (CCLE), Genomics of Drug Sensitivity in Cancer (GDSC), Achilles, and the MD Anderson Cell Lines Project (MCLP) [26–29]; a much more challenging setting would be to perform such integration when the different data sources are sensitive patient data held by different entities (research institutions, hospitals, companies, etc.), and the computations are first distributed among them and then brokered into a secure cloud resource.

## Conclusions

We describe the concepts behind the Patient Dossier, proposed as an interface between those who design innovative healthcare services, those who implement the technological building blocks, those who deal with legal and ethical implications, and those who look for business opportunities with the final users, clinicians, biomedical scientists, and possibly patients in mind. The point of communication is the actual questions that are asked about a patient, adapting the paradigm of software design in which an application programming interface (API) is used as a “contract” between different teams.

By laying out our vision of the Patient Dossier, we hope to provide a framework to communicate ideas and challenges between different communities with technologies that are largely available in the field. To exemplify these concepts, we describe an initial implementation of the Patient Dossier for handling genomic information in a Ruby framework called Rbbt.
